# Supplementary material for: Predictors of fear of falling among community-dwelling older adults: Cross-sectional study from Palestine
Source: PLoS One. 2022 Nov 17;17(11):e0276967. doi: 10.1371/journal.pone.0276967 (PMC9671337; doi:10.1371/journal.pone.0276967)
Supplement: Attachment — Submitted filename: Response to reviewers.docx [file pone.0276967.s002.docx]

Editor-in-Chief for PLOS ONE

Dear Editor

Greetings

We would like to submit the revised files of our manuscript Title: **Predictors of fear of falling among community-dwelling older adults: cross-sectional study from Palestine.**

Submission ID: PONE-D-21-23236

We did all the corrections suggested by the reviewers attached to this letter in point-to-point form, All the corrections have been approved by all authors. The changes in the manuscript were mentioned in the below table also they were highlighted in the manuscript for easier tracing.

Corresponding author:

Dr. Manal Badrasawi,

Email: [m.badrasawi@najah.edu](mailto:m.badrasawi@najah.edu)

Department of Nutrition and Food technology, Faculty of Agriculture and Veterinary Medicine, An-Najah National University, Tulkarm, West Bank PO. Box 7, Palestine

Thank you for your support.

Best Regards

| Reviewer | Comment Title | Comments | Reply | Changes in the manuscript |
| --- | --- | --- | --- | --- |
| Reviewer 1 |  | The authors looked into the relationship between fear of falling and several multi-domain parameters in Palestinian older adults. The manuscript is generally well written. The authors are to be commended for that. Though the topic is not novel, it could be potentially interesting for the readers of PLOS ONE. However, some issues are to be addressed before the manuscript can be accepted for publication. | Thank for your comment |  |
|  | Abstract | 1. “Fear of falling is a major health problem....”. This is an exaggeration. FOF has serious implications for health, which can be major health problems, but that’s just an indirect relationship. Please rephrase. | Ok | We rephrased it again (highlighted in yellow) |
|  |  | 2. Several scientific journals from the field or gerontology and geriatrics either advice again or even prohibit using the word “elderly” in submitted manuscripts, as this is a pejorative term. Please use “older adults” or other terms instead throughout the manuscript. | Ok | We used the term older adults in the entire manuscript (highlighted in yellow) |
|  |  | 3. “The Falls Efficacy Scale-International in order to predict falls among Palestinian older adults”: The sentence is incomplete. Please rephrase. | Ok | The Falls Efficacy Scale-International was also used to predict falls among Palestinian older adults |
|  |  | 4. At the results section the Timed Up-and-Go test is mentioned, although it wasn’t listed in the methods section. (Note that the TUG test does not fall under the category “ADL or IADL) | Ok | We added the following sentence in the methodology part (highlighted in yellow)   - and physical fitness performance (using senior fitness test).   We added the following sentence in the results part (highlighted in yellow):-   - Functional status (ADL, IADL), physical fitness status (timed up and go), and depression symptoms were significantly related to fear of falling. |
|  | Introduction | 5. “occasional fall”: Do you mean maybe “accidental fall”? | Yes | We changed it to accidental fall for better understanding (highlighted in yellow) |
|  |  | 6. “… a ground of high rates…”: what does this mean? Please rephrase |  | We changed it to “Falls are major causes of high rates of….” |
|  |  | 7. There are plenty of previous studies assessing the relationships between fear of falling and various multidisciplinary factors (partly the exact same with the ones used in this study); many of them even using a more robust design (longitudinal studies)*, which is more appropriate to define an outcome`s predictors. What is the exact research gap the authors try to address with this study? Does it have to do with the ethinicity of this sample? If so, how are Palestinians different from the samples of previous studies? Are different results expected? *e.g: Austin, N., Devine, A., Dick, I., Prince, R., & Bruce, D. (2007). Fear of falling in older women: a longitudinal study of incidence, persistence, and predictors. Journal of the American Geriatrics Society, 55(10), 1598-1603. Rivasi, G., Kenny, R. A., Ungar, A., & Romero-Ortuno, R. (2020). Predictors of incident fear of falling in community-dwelling older adults. Journal of the American Medical Directors Association, 21(5), 615-620. |  | We added the following highlighted in yellow  Given the paucity of studies regarding fear of falling among older adults in the Arab world. Therefore the main aim in the current study was to estimate the prevalence of fear of falling among older adults living in Hebron district, Palestine. We also hypothesized that parameters such as sociodemographic characteristics &lifestyle habits, medical history, body composition &anthropometric measurements, nutritional status, dietary practices, cognitive function, and physical functional status are associated with fear of falling, as previous literature indicated that they are related to aging process and age-related diseases. In the future, we are aiming to conduct longitudinal studies among older adults |
|  |  | 8. What is the reason for choosing the selected parameters as predictors of FOF in this study? | Previous literature indicated that these parameters are related to aging process, and age related disorders. so we hypothesized that they are may be related FOF | We added the following (highlighted in yellow)  We also hypothesized that parameters such as sociodemographic characteristics &lifestyle habits, medical history, body composition &anthropometric measurements, nutritional status, dietary practices, cognitive function, and physical functional status are associated with fear of falling, as previous literature indicated that they are related to aging process and age-related diseases. In the future, we are aiming to conduct longitudinal studies among older adults |
|  | Methods | 9. How can the authors be sure that this was a “representative” sample of Palestinian older adults?. |  | We added the following (highlighted in yellow): -  Palestine is considered a homogeneous country, as it is a small country divided into three regions (south, center, and south) that share the same traditions and food culture. So we selected Hebron district by stratified sampling method. Then, we randomly selected three different areas from Hebron district by random sampling. |
|  |  | 10. Why wasn’t the sample size calculation based on the regression analysis, which was also the main analysis of the study? | Ok, we recalculated using logistic regression model | We used G power software with an alpha of 0.05 (two-sided), CI of 95%, 80% power, and seventeen predictors based on difference between two independent proportions. A minimum of 206 participants was needed to determine the difference between the four groups (no fall concern, low fall concern, moderate and high fall concern) using logistic regression model. |
|  |  | 11. The last paragraph of the “sample size calculation and sampling method” is a bit confusing (mostly linguistically). Please rephrase. | Ok | We rephrased it (highlighted in yellow)  Palestine is considered a homogeneous country, as it is a small country divided into three regions (south, center, and south) that share the same traditions and food culture. So we selected Hebron district by stratified sampling method. Then, we randomly selected three different areas from Hebron district by random sampling. We used G power software with an alpha of 0.05 (two-sided) and 90% power based on difference between two independent proportions. A minimum of 186 participants was needed to determine the difference between the four groups (no fall concern, low fall concern, moderate and high fall concern). After considering 10% drop out and missing data, the sample size was increased to 200 participants. |
|  |  | 12. Why were nutritionists selected as an appropriate profession to conduct motor functioning geriatric assessments? | This study was done by a research team consisted of nutritionists who were interested in geriatric health and affiliated to nutrition department at the university. The research team also consisted of one physiotherapist who was trained to perform motor functioning assessment for older adults. | We added the following ( highlighted in yellow)  Data collection was done face-to-face throughout a research team consisted of two trained nutritionists to take anthropometric measurements and assess body composition, and one physiotherapist to perform physical function tests. |
|  |  | 13. There are several typos/language errors throughout the manuscript. Please make a careful check before re-submission | Ok | The manuscript was revised by an editor |
|  |  | 14. What was the rationale for building two categories of people based on their sleeping duration/problems? What does that have to do with FOF and the overall aims of the study and analysis? And what is the classification based on? Please explain and also provide references. | One of our aims in this study is to explore whether lifestyle factors (e.g. sleeping duration, etc.) associated with fear of falling among older adults, moreover, there a hypothesis states that sleeping duration/ sleeping problems affects the balance and alertness in older adults. Therefore we categorized participants into two categories according to their sleeping duration/ sleeping problems. |  |
|  |  | 15. “Many screening instruments are available to assess concerns about falling among elderly people”: I am not familiar with any other besides the FES. Can you please name some? | Ok | We added the following (highlighted in yellow), and we also added references (highlighted in yellow)  Many screening instruments (e.g., Adapted Falls Efficacy Scale “Adapted FES” [Tiernan et al., 2014],Modified Falls Efficacy Scale “MFES” [Akosile et al., 2014], Concern and Fear about falling “CAFlik” [Valentine et al., 2010], Activities-specific balance confidence scale “ABC scale” [Jones et al., 2006], Survey of Activities and Fear of Falling in the Elderly “SAFFE/SAFE” [Yeung et al., 2006], and Fear of falling subscale “FOF subscale” [Warnke et al., 2004]) are available to assess concerns about falling among older adults.. |
|  |  | 16. The section “nutritional status” barely contains anything related to nutrition. A title like “Body Composition” seems more appropriate. | Ok | Highlighted in yellow |
|  |  | 17. “Cognitive function was assessed using a validated Arabic version of the short geriatric depression scale (GDS-15) in order to detect depression among older adults”, and “The presence of depressive symptoms was assessed using a validated Arabic version of Montreal cognitive assessment –basic to detect mild cognitive impairment.”: Depression and cognition may be often related but there are completely different constructs. Please explain what you mean. | Matching error | We changed it (highlighted in yellow)  A validated Arabic version of the short geriatric depression scale (GDS-15) was used to detect depression among older adults.  Moreover, a validated Arabic version of Montreal cognitive assessment – basic to detect mild cognitive impairment [19]. |
|  |  | 18. ADL and iADL are highly correlated. Why use both as possible predictors and have you checked for multicollinearity? |  | Thank you for the comments. Only IADL has been added in the regression model as one of the independent variables. The multicollinearity has been determined using the VIF value in linear regression.  This has been added in the statistical analysis section |
|  | Results | 19. Figures 1 and 2 are a too simplistic and in my opinion unnecessary. The information presented there can be easily integrated into the text (which was partly already done-in figure 2)). | Ok | We removed figure 1 and 2 |
|  |  | 20. Please discuss the unbalance between the number of men and women in the sample and how this can affect the results. | In Palestinian culture, the majority of older women stay at their homes as they are responsible for household chores. In contrast, most older men stay out of their homes, as they are either employees or have their private projects. That’s mean that the majority of participants were older adults as they were available at their homes during data collection process.  The unbalance between males and females don’t affect the results. |  |
|  |  | 21. The descriptive results take up most of the results section. I suggest to shorten and focus on the associations. | Ok | We removed a large part of descriptive results and we displaced it with the following sentence Participants’ demographic characteristics, nutritional status, cognitive, and physical functional status are presented in Table 1, and Table 2, respectively. |
|  |  | 22. “Table 3 shows the relationship between…..”: This table presents descriptive results (means). In what way can relationships be derived from this table? | Data are presented as n (%) or mean ± SD; * p < 0.05.  Pearson [chi-square test](https://www.sciencedirect.com/topics/medicine-and-dentistry/chi-square-test) is employed for categorical variables and Kruskal-Wallis test for continuous variables. | The following is written under the table (highlighted in yellow)  Data are presented as n (%) or mean ± SD; * p < 0.05.  Pearson [chi-square test](https://www.sciencedirect.com/topics/medicine-and-dentistry/chi-square-test) is employed for categorical variables and Kruskal-Wallis test for continuous variables. |
|  | Discussion | 23. Why was such focus given on nutrition (in the discussion but also throughout the manuscript) when nutrition is just one of the many factors that potentially relate to FOF? Following up on this point, why was nutrition even selected as a factor? Please explain the direct (if it exists) relationship between nutrition and FOF |  | We added the following (highlighted in yellow), and we also added references (highlighted in yellow)  Yardley and Smith reported that FOF is correlated with poorer health and balance, indicating a negative effect of FOF to balance [Yardley &Smith. 2009]. Shahar and his colleagues, for their parts, revealed that good nutritional status improves both functional abilities and balance abilities among older adults [Shahar et al., 2009]. Moreover, there is evidence that poor nutritional status results in muscle weakness and low muscle mass, which in turn leads to impaired mobility and the necessity for using ambulatory aids like walker [De Guzman et al., 2013]. However, studies in relation to nutritional status and FOF are still scarce. |
| Reviewer 2 |  | Please use the term older adults instead of elderly | Ok | Highlighted in yellow |
|  | Abstract | Present first the assessment used for fall, as it is the main variable of the study. | ok | Highlighted in yellow |
|  |  | Present the % of men, women | Ok | We added the following sentence (highlighted in yellow)   - 137 (68.5%) females and 63 (31.5%) males. |
|  |  | Present statistical results to support the findings | Ok | We added the p-value (highlighted in yellow) |
|  |  | Conclusion, does not make the responsibility for healthcare professionals and yes tell about policies and procedures. Because this is a correlational study, conclude using this aspect and not about interventions. | Ok | We added the following (highlighted in yellow)  High concern of falling is significantly associated with advanced age, low education level, being married, and living in villages or camps. ADLs were among the factors that had a significant relationship with increased fear of falling. Predictors of fear of falling among Palestinian older adults were IADL scores, body fat percentage, rapid gait speed, timed up and go test. Future studies could investigate further correlates of fear of falling among older adults. |
|  | Introduction | The introduction presents important information. Because this could be one of the first study in Palestine (it is not possible to confirm as not all studies are available online and in databases), the authors could explain what the particular relevance of this study for the Palestinian population and healthcare are. Also, do not forget to identify the missing in the literature this study wants to cover and the state the study-question. Do not forget to link the conclusion section with the study question. |  | We added the following highlighted in yellow  Given the paucity of studies regarding fear of falling among older adults in the Arab world. Thus, our main aim in the current study is to estimate the prevalence of fear of falling among older adults living in Hebron district, Palestine, and to identify its' associated risk factors, including; sociodemographic characteristics &lifestyle habits, medical history, body composition &anthropometric measurements, risk of malnutrition, dietary practices, cognitive function, and physical functional status. In the future, we are aiming to conduct longitudinal studies among older adults |
|  | Methods | (please use Methods and not Methodology) | Ok | Highlighted in yellow |
|  |  | Report fear of falling first | Ok | We reported first (highlighted in yellow) |
|  |  | For the heading of Cognitive function, change for Depressive Symptoms and Cognitive function as you are presenting GDS and MOCA. | Ok | Highlighted in yellow |
|  |  | Statistical analysis section needs to be expanded, giving more details, in special about the regression analysis. This should be as a receipt, telling step by step, you can refer the Tables and figures in the results. |  | Thank you for the comments. The statistical analysis section in the methodology section has been expanded based on the results of the study. The multicollinearity assumption has been added in the explanation of regression model |
|  | Results | All the topics about patients’ recruitment should appear in the Methods section and not Results. | Ok | We moved participants’ recruitment paragraph to methods section (highlighted in yellow) |
|  |  | Tables need to be formatted |  |  |
| Reviewer 3 |  | Thank you for inviting me to review this manuscript. This study is about falls risk predictors among community-dwelling older adults in Palestine. Although well-intended, the study has shortcomings that limit my enthusiasm. |  |  |
|  |  | A first comment is for the (in)congruence between the title – “Predictors of fall risk…” and the aims of the study - “… to investigate risk for fear of falling among Palestinian older adults….” . Risk of falls and risk for fear of falling are conceptually different. Fear of falling can be a determinant of risk of falls and also a consequence of falls. So, the authors need to be clearer about the differences. In fact, when I read the manuscript, it seems that the authors intended to explore factors related to fear of falling, rather than to risk of falling. In this sense, please elaborate more on the relation about predictors of fear of falling -fear of falling – and risk of falling. | Typing error | Corrected in the entire manuscript (highlighted in yellow) |
|  |  | Moreover, predictors of falls or predictors of fear of falling are well established in the literature, thus I cannot see what the novelty of this study is, beside cultural issues, like being conducted among Palestinian older population. If this is the case, the authors should address how cultural issues might have impacted the results in the discussion section. |  | We added the following highlighted in yellow  Given the paucity of studies regarding fear of falling among older adults in the Arab world. Therefore the main aim in the current study was to estimate the prevalence of fear of falling among older adults living in Hebron district, Palestine. We also hypothesized that parameters such as sociodemographic characteristics &lifestyle habits, medical history, body composition &anthropometric measurements, nutritional status, dietary practices, cognitive function, and physical functional status are associated with fear of falling, as previous literature indicated that they are related to aging process and age-related diseases. In the future, we are aiming to conduct longitudinal studies among older adults |
|  | Introduction | In the Introduction, the background needs to be updated regarding epidemiological data about the world aging population, as there are recent references about demographic projections besides that of Newgarde et al. 2013. | Ok | We added the following sentence in the introduction part (highlighted in yellow)  WHO reports indicates that the world aging population was 1 billion in 2019, and this figure is expected to rise to 2.1 billion by 2050 [1].  The reference (highlighted in yellow):-  World Health Organization. Ageing and Health. 2022. http:// [www.who.int/health-topics/ageing#tab=tab_1](http://www.who.int/health-topics/ageing#tab=tab_1). Assessed on 20.05.2022. |
|  |  | Since FES-I is the instrument used to assess the variable of interest in this study, the authors also need to revise the different constructs of fear associated with falls and revise which construct they really are aiming to assess – Concern about falling. For inspiration read this article: J Aging Health. 2011 Feb;23(1):3-23. doi: 10.1177/0898264310378039. Epub 2010 Sep 17. Reconceptualizing the role of fear of falling and balance confidence in fall risk. Hadjistavropoulos T1, Delbaere K, Fitzgerald TD. |  | The following has been added (highlighted in yellow)  Evaluating all of the three components of fear of falling (i.e., behavioral, physiological, and cognitive) could lead to more precise prediction of fall risk [Hadjistavropoulos et al., 2010]. Self-efficacy beliefs are associated with fear of falling as fear is influenced by beliefs. Hadjistavropoulos and his colleagues (2010) indicated that there is a relationship between the two constructs “fear of falling and self-efficacy”, however, they differ from each other. Over the years, many assessment tools have been used to access falls efficacy and fear of falling. The most commonly used tool is the Falls Efficacy Scale – International “FES-I”, which consists of 10 items from the original scale and 6 items on social activities (e.g., going to a social event, visiting relatives and friends), and more complex instrumental activities of daily living “IADL” (e.g., ability to use telephone) [Yardley et al., 2005], have been found to be able to predict the risk of falls and to assess the level of falling concern. |
|  |  | A last comment regarding the background is related to the 4th paragraph. A more clear and sustained relation about the fear of falling and variables such as mobility, functional decline, depression, activity restriction, just to name a few, should be emphasised. |  | The following has been added highlighted in yellow  Gagnon and his colleagues argued that activity avoidance (often developed as a response of fear of falling), raises the risk of fall, functional decline, and pain, since it can result in deconditioning (e.g., immobility, imbalance, lowered function, strength), changes in gait, and poor balance [Gagnon et al., 2005]. Moreover, exaggerated activity avoidance can lead to reduced ability to perform activities of daily living (ADLs). Models that make such presumption have been illustrated as fear-avoidance models [e.g., Hadjistavropoulos et al., 2010].  Evidence also suggested that older adults who have fear of falling show longer double support time, slower gait speed, and shorter stride in comparison to their counterparts [Chamberlin et al., 2005]. In addition, fear of fall is correlated with gait variability, which is viewed as fall predictor [Sawa et al., 2014]. |
|  | Methods | The methods section is not presented in a standard way: Study design, Participants, Data collection; data analysis. | Thank you for the comment  We revise the methodology section, and we modify it according to STROBE ( attached is the checklist for the STROBE guidelines_ below this table) |  |
|  |  | Regarding exclusion criteria, how was “having dementia” assessed? Were participants allowed to use walking aid? | After we get the written consent from participants (older adults), we asked their caregivers if they have either mobility problems or dementia.  The participants were asked to perform the test as they usually walk. And if they use walking aid they were asked to use it |  |
|  |  | Did participants report a history of falls in the last 3, 6 or 12 months? |  | The following sentence has been added (highlighted in yellow)  According to FES-I protocol, older adults were also asked if they had a history of fall within the past six months. |
|  |  | Please provide cut-off scores for the IPAQ and interpretations. | Ok | We added the following (highlighted in yellow)  Metabolic equivalent. minutes per week (MET.min/week) for each of walking, moderate- and vigorous-intensity activities according to IPAQ scoring protocol [<http://www.ipaq.ki.se>]. A score of ≤ 3 MET indicates low level of physical activity, a score of 3 -6 MET indicates moderate level of physical activity, and a score of ≥ 6 MET indicates a high level of physical activity [Ainsworth et al., 2000]. |
|  |  | Please go into more detail regarding the psychometric properties of the instruments used (e.g., FES-I, MOCA, GDS, TUG, etc.) | Ok | The following was added (highlighted in yellow):-   - We have performed clinical assessment of participants’ nutritional status by using a validated screening tool named Mini Nutritional Assessment-Short form-Arabic Version (MNA-SF-A). A previous study showed that the original scale is valid with sensitivity equal to 97.9% and specificity of 100% [Rubenstein et al., 2001]. - A validated Arabic version of the short geriatric depression scale (GDS-15), which has a high sensitivity (83%) and specificity (91%), - Moreover, a validated Arabic version of Montreal cognitive assessment – basic was used to detect mild cognitive impairment [Rahman &El Gaafary. 2009]. This tool has a sensitivity of 92.3% and specificity of 85.7% [Rahman &El Gaafary. 2009]. - The ADL, which has a sensitivity of 38% and a specificity of 80%, was assessed using the Katz index scale [Hartigan. 2007]; and the IADL, which has a sensitivity of 62% and a specificity of 80% at the lowest cut-off point for the diagnosis of cognitive impairment, was assessed using the Lawton scale [Lawton &Brody. 1969]. |
|  |  | GDS does not assess cognitive function but depressive symptoms. Please correct this information for DGS and MOCA | Ok | Corrected and highlighted in yellow (A validated Arabic version of the short geriatric depression scale (GDS-15) was used to detect depression among older adults) |
|  | Results | In the results section, there are too many tables and figures that are not easy to follow. | Ok | We removed figure 1 and figure 2 |
|  |  | Please provide a table describing the sample’s sociodemographic, without separating women and man. It is not clear why the authors present data for men and women separately without any statistical analysis between them in Table 1. |  | Ok, we presented the data as a total sample (highlighted in yellow) |
|  |  | Finally, all the English must be carefully revised. | Ok | The manuscript was revised by an editor |

STROBE Statement—Checklist of items that should be included in reports of ***cross-sectional studies***

|  | Item No | Recommendation |
| --- | --- | --- |
| **Title and abstract** | 1 | (*a*) Indicate the study’s design with a commonly used term in the title or the abstract |
|  |  | (*b*) Provide in the abstract an informative and balanced summary of what was done and what was found |
| Introduction | | |
| Background/rationale | 2 | Explain the scientific background and rationale for the investigation being reported |
| Objectives | 3 | State specific objectives, including any prespecified hypotheses |
| Methods | | |
| Study design | 4 | Present key elements of study design early in the paper |
| Setting | 5 | Describe the setting, locations, and relevant dates, including periods of recruitment, exposure, follow-up, and data collection |
| Participants | 6 | (*a*) Give the eligibility criteria, and the sources and methods of selection of participants |
| Variables | 7 | Clearly define all outcomes, exposures, predictors, potential confounders, and effect modifiers. Give diagnostic criteria, if applicable |
| Data sources/ measurement | 8* | For each variable of interest, give sources of data and details of methods of assessment (measurement). Describe comparability of assessment methods if there is more than one group |
| Bias | 9 | Describe any efforts to address potential sources of bias |
| Study size | 10 | Explain how the study size was arrived at |
| Quantitative variables | 11 | Explain how quantitative variables were handled in the analyses. If applicable, describe which groupings were chosen and why |
| Statistical methods | 12 | (*a*) Describe all statistical methods, including those used to control for confounding |
|  |  | (*b*) Describe any methods used to examine subgroups and interactions |
|  |  | (*c*) Explain how missing data were addressed |
|  |  | (*d*) If applicable, describe analytical methods taking account of sampling strategy |
|  |  | (*e*) Describe any sensitivity analyses |
| Results | | |
| Participants | 13* | (a) Report numbers of individuals at each stage of study—eg numbers potentially eligible, examined for eligibility, confirmed eligible, included in the study, completing follow-up, and analysed |
|  |  | (b) Give reasons for non-participation at each stage |
|  |  | (c) Consider use of a flow diagram |
| Descriptive data | 14* | (a) Give characteristics of study participants (eg demographic, clinical, social) and information on exposures and potential confounders |
|  |  | (b) Indicate number of participants with missing data for each variable of interest |
| Outcome data | 15* | Report numbers of outcome events or summary measures |
| Main results | 16 | (*a*) Give unadjusted estimates and, if applicable, confounder-adjusted estimates and their precision (eg, 95% confidence interval). Make clear which confounders were adjusted for and why they were included |
|  |  | (*b*) Report category boundaries when continuous variables were categorized |
|  |  | (*c*) If relevant, consider translating estimates of relative risk into absolute risk for a meaningful time period |
| Other analyses | 17 | Report other analyses done—eg analyses of subgroups and interactions, and sensitivity analyses |
| Discussion | | |
| Key results | 18 | Summarise key results with reference to study objectives |
| Limitations | 19 | Discuss limitations of the study, taking into account sources of potential bias or imprecision. Discuss both direction and magnitude of any potential bias |
| Interpretation | 20 | Give a cautious overall interpretation of results considering objectives, limitations, multiplicity of analyses, results from similar studies, and other relevant evidence |
| Generalisability | 21 | Discuss the generalisability (external validity) of the study results |
| Other information | | |
| Funding | 22 | Give the source of funding and the role of the funders for the present study and, if applicable, for the original study on which the present article is based |

*Give information separately for exposed and unexposed groups.

**Note:** An Explanation and Elaboration article discusses each checklist item and gives methodological background and published examples of transparent reporting. The STROBE checklist is best used in conjunction with this article (freely available on the Web sites of PLoS Medicine at http://www.plosmedicine.org/, Annals of Internal Medicine at http://www.annals.org/, and Epidemiology at http://www.epidem.com/). Information on the STROBE Initiative is available at www.strobe-statement.org.
