# Supplementary material for: Predictors of fear of falling among community-dwelling older adults: Cross-sectional study from Palestine
Source: PLoS One. 2022 Nov 17;17(11):e0276967. doi: 10.1371/journal.pone.0276967 (PMC9671337; doi:10.1371/journal.pone.0276967)
Supplement: Attachment — Submitted filename: PONE.docx [file pone.0276967.s003.docx]

Overall, this is a well-written manuscript of a well-designed study. I am highlighting the areas in which I believe the manuscript could be clarified or improved.

Abstract:

1. For abbreviations and acronyms spell out the full term at its first mention, like ADL, IADL

Introduction:

-Please add appropriate reference in the first paragraph

- I would suggest adding a paragraph talking about falls and fear of falling in the Middle east, and emphasize how serious and prevalent is fear of falling. ( suggested articles DOI: [10.1016/j.archger.2019.04.006](https://doi.org/10.1016/j.archger.2019.04.006), DOI: [10.1111/ajag.12673](https://doi.org/10.1111/ajag.12673) , DOI: [10.1016/j.archger.2018.08.001](https://doi.org/10.1016/j.archger.2018.08.001)

Methods:

- In the method section, the author should report whether there is any data missing and how missing data is processed. Also, add a flow chart of the participants selection.
- Use “and” instead of using “&”
- History of falls has been consistently reported as a risk factor of fear of falling in previous studies. Have taken it into consideration if not, why?
- In your inclusion criteria of age was 60 years and older, and yet in your sample the age ranges from 65-98. Please explain?
