# Supplementary material for: Predictors of fear of falling among community-dwelling older adults: Cross-sectional study from Palestine
Source: PLoS One. 2022 Nov 17;17(11):e0276967. doi: 10.1371/journal.pone.0276967 (PMC9671337; doi:10.1371/journal.pone.0276967)
Supplement: Attachment — Submitted filename: point to point letter.docx [file pone.0276967.s004.docx]

Editor-in-Chief for Plos one

Dear Editor

Greetings

We would like to submit the revised files of our manuscript Title: **Predictors of fear of falling among community-dwelling older adults: cross-sectional study from Palestine.**

We did all the corrections suggested by the reviewers we did them in point to point table. All the corrections have been approved by all authors. The changes in the manuscript were mentioned in the below table also track changes was used to highlight changes.

Corresponding author:

Dr. Manal Badrasawi,

Email: [m.badrasawi@najah.edu](mailto:m.badrasawi@najah.edu)

Department of Nutrition and Food technology, Faculty of Agriculture and Veterinary Medicine, An-Najah National University, Tulkarm, West Bank PO. Box 7, Palestine

Thank you for your support.

Best Regards

|  | Comment | Reply | Changes on the manuscript |
| --- | --- | --- | --- |
| Editor comments | You did a good job on responding to the previous reviewers comments and suggestions. However, since the previous reviewers were not responsive, we had to send it to other reviewers. Please, revise according to their suggestions and submit it again. | Thank you |  |
|  | Please review your reference list to ensure that it is complete and correct | Done | Please refer to the manuscript file |
| Reviewer #6 | Overall, this is a well-written manuscript of a well-designed study. I am highlighting the areas in which I believe the manuscript could be clarified or improved. |  |  |
|  | Abstract: For abbreviations and acronyms spell out the full term at its first mention, like ADL, IADL | Corrected  Added as full term in the first time | and functional status (using activities of daily living and instrumental activities of daily living scale), |
|  | Introduction:  -Please add appropriate reference in the first paragraph  - I would suggest adding a paragraph talking about falls and fear of falling in the Middle east, and emphasize how serious and prevalent is fear of falling. ( suggested articles DOI: [10.1016/j.archger.2019.04.006](https://doi.org/10.1016/j.archger.2019.04.006), DOI: [10.1111/ajag.12673](https://doi.org/10.1111/ajag.12673) , DOI: [10.1016/j.archger.2018.08.001](https://doi.org/10.1016/j.archger.2018.08.001) | Done  Thank you, the references were added | In general, this elevation in the relative percentage in comparison to the overall population has great implications both for health services and for older adults [1].  In a systematic review, included six studies conducted among Arabic population, Gulf Cooperation Council Countries, the pooled prevalence of fall was 46.9%ranged from 34% to 57.7% [9]. While in Turkey, the prevalence of falls among older adults aged 80 years and above was 35.4%, and fear of falling was 86.6% [10]. Among Iranian population, the prevalence of falls was 39.7% among 653 older adults [11].  The references were also added to the references list |
|  | Method  In the method section, the author should report whether there is any data missing and how missing data is processed. Also, add a flow chart of the participants selection.  Use “and” instead of using “&”  History of falls has been consistently reported as a risk factor of fear of falling in previous studies. Have taken it into consideration if not, why?  In your inclusion criteria of age was 60 years and older, and yet in your sample the age ranges from 65-98. Please explain? | There were 10 participants who were excluded due to missing data  We added the flow chart in the primary submission  Please see attached figure below the table    Corrected  We agree with this valuable comment. We have the data for history of fall and we are currently writing a manuscript having the history of fall as main variable.  The inclusion criteria was 65  We correct it | Please refer to the first submission  Please refer to the manuscript  No change in the manuscript  In the current study, participants were included if they aged 65 years old or above, and willing to participate and to provide all the required data. |
| Reviewer #4 | Fear of falling and its predictors among older adults is an important topic. Exploring those factors in specific community would help health care providers detect fear of falling early on and address them before getting serious injuries.  Authors of this manuscript did a great job in investigating several important factors and analyze the results in the appropriate statistics. | Thank you |  |
| Reviewer #5: | 1. Under Sample size and sampling techniques: a small country divided into three regions (south, center, and south) need to correct it.  2. Reference: there is differences in the reference style | Corrected  All the reference were revised and corrected | (north, center, and south) |
